# Remarks from the Editor-in-Chief

**DOI:** 10.1017/psy.2025.10076

**Published:** 2026-01-07

**Authors:** Sandip Sinharay

**Affiliations:** ETS Research Institute

Dear *Psychometrika* Readers,

Welcome to the fifth and final Psychometrika issue of 2025. If you are wondering if this is really the “fifth” issue of the year, I would like to remind you that Psychometrika now publishes five issues per year.

This Psychometrika issue starts with an obituary. Robert Mislevy, a past president of the Psychometric Society and a Psychometric Society career award winner, unexpectedly passed away in May 2025. His former colleagues, Roy Levy and Russel Almond, teamed up to write an obituary of Prof. Mislevy that I highly recommend to all of you so that you are able to know more about the life and work of this extraordinary scholar and gentleman. This issue then includes twelve “Theory and Methods” section articles. In the first of these, Weicong Lyu, Chun Wang, and Gongjun Xu suggest a novel regularization method for detecting differential item functioning in item response theory models. The second, by Sunbeom Kwon and Susu Zhang, presents a latent class mediation analysis procedure for explaining performance gaps with examinee process data. In the third article, Jia Liu, Seunghyun Lee, and Yuqi Gu propose a general modeling framework featuring a highly flexible data layer that is adaptive to various response types and measurement models for cognitive diagnostic models. The fourth “Theory and Methods” section article, by L. Andries van der Ark, provides analytic nonparametric standard errors for coefficients used in reliability analysis. The fifth article, by Seewoo Li and Hyo Jeong Shin, proposes a novel item response theory model to handle continuous responses and sparse polytomous responses in psychological and educational measurement. Two articles on factor analysis follow. In the first of these, Ryoya Fukasaku, Kei Hirose, Yutaro Kabata, and Keisuke Teramoto propose an algebraic approach to compute all candidates for the maximum likelihood estimate in a factor-analysis problem; the second, by Nanyu Luo and Feng Ji, proposes importance-weighted adversarial variational Bayes for high-dimensional item factor analysis. The eighth “Theory and Methods” section article, by Martin Papenberg, Martin Breuer, Max Diekhoff, Nguyen K. Tran, and Gunnar W Klau, investigate if the bicriterion approach for anticlustering can be improved using exact algorithms that guarantee globally optimal criterion values. In the ninth article, Maarten Marsman, Lourens Jan Waldorp, Nikola Sekulovski, and Jonas Haslbeck suggest a Bayesian framework for the analysis of ordinal Markov random fields, a network model for binary and ordinal variables, and develop Bayes-factor tests for assessing parameter differences in the networks of two independent groups. In the tenth article, Valerii Dashuk, Martin Hecht, Oliver Lüdtke, Alexander Robitzsch, and Steffen Zitzmann propose an optimally regularized Bayesian estimator of multilevel latent variable models that aims to outperform traditional maximum likelihood estimation in mean squared error performance. The eleventh article, by Wataru Urasaki, Tomoyuki Nakagawa, Jun Tsuchida, and Kouji Tahata, proposes an approach to correspondence analysis for evaluating departures from symmetry in square contingency tables with nominal categories, using a modified divergence statistic. In the last “Theory and Methods” section article, Daniel C. Furr and Jianbin Fu propose a ranking pattern approach to build item response theory models for forced-choice items.

This Psychometrika issue then includes three book reviews. In the first of these, Jordan Rickles reviews the 2024 book “A First Course in Causal Inference” written by Peng Ding. The second book review, written by Edanur Dayıoğlu, focuses on the 2024 book “Complex-Systems Research in Psychology” by Han L. J. van der Maas. In the third, Geoffrey Blondeller reviews the 2023 book “An Introduction to Psychometrics and Psychological Assessment (2nd Edition)” written by Colin Cooper.

This issue ends with an erratum, written by Giuseppe Mignemi and Ioanna Manolopoulou, on their 2025 Psychometrika article “Bayesian Nonparametric Models for Multiple Raters: A General Statistical Framework.”

Hope you enjoy the issue.

